# Antibody response to pneumococcal conjugate vaccine 10 among Nigerian children under 5 years

**DOI:** 10.4314/ahs.v23i2.20

**Published:** 2023-06

**Authors:** Chidinma A Udah, Francis U Iregbu, Emmanuel E Ekanem

**Affiliations:** 1 Department of Paediatrics, Federal University Teaching Hospital, Owerri; 2 Department of Paediatrics, University of Calabar Teaching Hospital

**Keywords:** Pneumococcal conjugate vaccine, antibody, children, Nigeria

## Abstract

**Introduction:**

Invasive pneumococcal diseases have been a major contributor to childhood mortality, particularly in the developing world and pneumococcal vaccines were introduced to reduce the burden. The Pneumococcal Conjugate Vaccine 10 (PCV 10) was incorporated into the Nigerian National Programme on Immunization (NPI) in 2014 to reduce the incidence of childhood pneumococcal infections. This study was done to determine the immunogenicity of the vaccine in our clime.

**Methodology:**

This cross-sectional study was carried out between September 2019 and January 2020 at the Children Outpatient Clinic of the Federal Medical Center, Owerri, Nigeria. Two hundred and forty five children between the ages of 20 weeks and 59 months, who had received three doses of Pneumococcal Conjugate Vaccine 10 (PCV 10) at 6, 10 and 14 weeks according to the NPI schedule, were recruited into the study. The anti-pneumococcal PCV 10 IgG concentration was determined using Human Anti-Pneumococcal CPS 10 IgG vaccine ELISA Kit ®. Simple proportions, means and median (as appropriate) were used to analyse the data. Kruskal Wallis test and Spearman's correlation were done to test association. Significance was set as p< 0.05.

**Results:**

The mean anti-pneumococcal IgG concentration was 11.01±1.23 IU/ml and all the study participants formed protective levels of anti-pneumococcal IgG. There was a slight positive correlation between antibody response and age (r=0.13, p=0.04), and the antibody response was slightly more in males than females.

**Conclusion:**

All the children under the age of five years who had received PCV 10 at 6, 10 and 14 weeks of age, who participated in this study formed protective levels of antibodies to the vaccine. Antibody levels increased slightly with age. The PCV 10 currently used in the Nigerian programme is sufficiently antigenic and a downward trend in pneumococcal diseases should soon be noticeable.

## Introduction

Streptococcus pneumoniae (pneumococcus) is a very important pathogen that is a major cause of global morbidity and mortality.^1^ The World Health Organization (WHO) estimates that 1.6 million people, including about one million children less than five years of age, die of pneumococcal infections annually, with developing countries bearing the greatest burden.^2^ The ten countries with the highest burden of pneumococcal infections are in Africa and Asia, and together they account for 66% of reported cases worldwide.^2^ Nigerian children made up the highest number of those who died, with an estimated 162,000 deaths in 2018, i.e. 443 deaths per day, or 18 every hour.3 In Nigeria, it was the commonest cause of mortality in under five children in 2017 and 19% of child deaths in 2018 were due to pneumonia.^3^ In developing countries, the pneumococcus is the most frequent cause of childhood pneumonia and the mortality from pneumococcal meningitis is high (approximately 50%) with many survivors left with severe neurologic disabilities.^4^,^5^ Given the emergence of widespread resistance to antibiotics, prophylactic vaccines were formulated on the basis of the most common capsular serotypes.^4^ Pneumococcal Conjugate Vaccines have been shown to reduce the incidence of invasive pneumococcal diseases, as demonstrated in the trial done by Cutts et al^6^ in Gambia. The nine-valent Pneumococcal Conjugate Vaccine yielded a 37% efficacy against the first episode of radiological pneumonia, 77% efficacy against invasive pneumococcal disease caused by vaccine serotypes, 50% against disease caused by all serotypes, and 15% against all-cause admissions.^6^ A population-based surveillance study in Gambia by Mackenzie et al^7^, following introduction of the vaccine,, showed similar results, which are in keeping with results from South Africa,^8^ and the United States of America.^9^

The Pneumococcal Conjugate Vaccine 10(PCV 10) was introduced into the Nigerian Programme on Immunization in December 2014,where children receive three doses of the vaccine at 6, 10 and 14 weeks of age without a booster dose.^10^ PCV 10 contains antibodies to serotype 1,4,5,6B,7F,14,18C,19F and 23F, which are the serotypes prevalent in the West African region.5 According to the WHO, the effectiveness of pneumococcal conjugate vaccines can be evaluated based on serum immunoglobulin G (IgG) levels.^11^ Odusanya et al^12^, in a randomized control trial done in Nigeria and Mali in 2014 observed that 98.5 % formed protective antibody levels, after PCV 10 immunization.^12^ Similar results were obtained by Dicko et al^13^ and Akinsola et al^14^. Since the launch of the PCV 10 vaccine in Nigeria in 2014,10 there has been a rising number of children enrolling, but there are few published studies on antibody response to the vaccine. This work was therefore designed to evaluate the immunogenicity of PCV 10 as one of the ways of determining the effectiveness of the vaccine in Nigerian children.

## Materials and methods

This was a cross sectional descriptive hospital-based study, carried out at the Children Outpatient Clinic (CHOP) of the Federal Medical Center Owerri, Nigeria, between September 2019 and January 2020. The study population consisted of children aged 5 to 59 months, who had received three doses of PCV 10 at 6, 10 and 14 weeks, at least 6 weeks prior, whose caregivers gave consent and showed evidence of immunization (the immunization card). Malnourished children and those who had chronic illnesses such as malignancies and tuberculosis were excluded from the study. The sample size of 245 was derived using the Cochran formula, and participants were recruited consecutively until the desired sample size was achieved. On recruitment, relevant information from the participants were obtained and entered into pre-tested interviewer administered proformas. Blood samples were collected using aseptic techniques and samples were stored in sample bottles at room temperature and subsequently transferred to the Laboratory within one hour of collection. Pooled serum samples were analysed using the Human Anti-S Pneumococcal vaccine (Synflorix/PCV-10) IgG ELISA kit® (Alpha Diagnostic Intl, USA) for quantitation of Anti-CPS IgG to 10 serotypes(1,4,5,6B, 7F,9V,14,18C,19F,23F) with strict adherence to the manufacturer's instructions and standard operating procedures.^15^ The protective anti-pneumococcal IgG level was set at 1 IU/ml according to the manufacturer's manual.^15^

### Ethical considerations

Ethical approval was obtained from the Health Research and Ethics Committee of the Federal Medical Center Owerri (FMC/OW/HREC/186). The purpose of the study was explained to the participants and written informed consent that adequately recorded the participant's informed decision was obtained from the parents/guardians before the commencement of the study. All information provided by the participants were kept confidential.

### Data Analysis

Data entry was done using an Excel sheet, it was checked for completeness and transferred to Statistical Package for Social Sciences (IBM SPSS statistics for Windows version 25.0, Armonk, NY: IBM Corp) for analysis. Simple proportions, means and standard deviations were used to analyse normally distributed data such as height/length and antibody concentration. Median and interquartile ranges were used to analyse non-normally distributed data such as weight.

Student t-test was used to compare the mean between two groups, while comparison of antibody response across more than two groups was done using analysis of variance (ANOVA). A Kruskal-Wallis test was used to compare non-normally distributed variables across three or more categories. Spearman's correlation analysis was used to determine the relationship between age and antibody levels. Statistical significance was set at p<0.05.

## Results

A total of two hundred and forty-five (245) children were recruited from the Children's Out-patient clinic (CHOP) into the study.

Table I shows the socio-demographic details of participants. The median age of participants was 20 months, with an interquartile range of 11-36 months. Age group 12-23 months was the most frequently studied with a frequency of 77 (31.4%). There were more males than females (56.3% vs 43.7%) and majority of participants resided in urban areas (73.5%).

**Table I:** Socio-demographic characteristics of participants

Variable	Frequency(n)	Percentage (%)
**Age (months)**		
Median (IQR) = 20(11-36)		
**Age group (months)**		
<12	63	25.70
12-23	77	31.40
24-35	40	16.30
36-47	32	13.10
48-59	33	13.50
**Sex**		
Female	107	43.70
Male	138	56.30
**Residence**		
Rural	65	26.50
Urban	180	73.50

The mean anti-pneumococcal IgG levels following three doses of PCV 10, among the study participants was11.01±1.23 IU/ml. The minimum IgG level was 3.84 IU/ml, while the maximum was 16.42 IU/ml.

**Figure 1:**
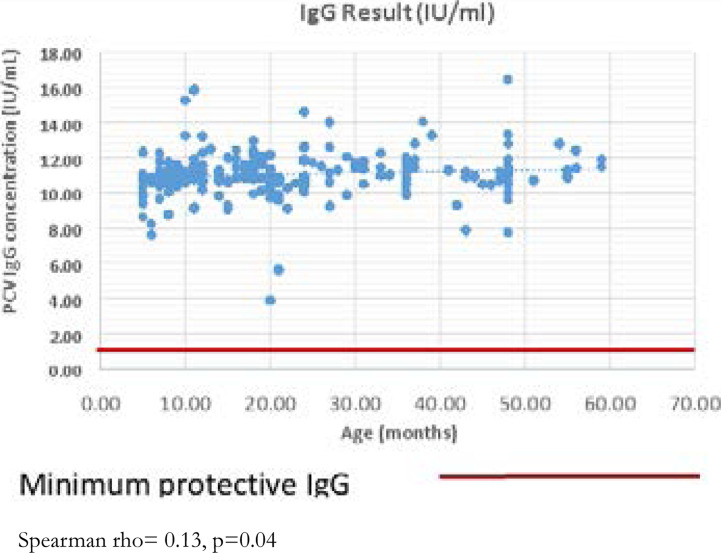
Correlation between age and anti-pneumococcal IgG concentration

One hundred percent of all the participants in the study formed protective levels of anti-pneumococcal IgG (> 1 IU/ml) following immunization with PCV 10 (Figure 1). The correlation between age and anti-pneumococcal IgG antibody levels was weakly positive (r=0.13) and statistically significant (p=0.04).

Table II shows the anti-pneumococcal IgG concentrations among the studied age groups. The antibody concentration was highest in the 48–59-month age group, however the difference between this value and that from the other groups was not statistically significant (p=0.22).

**Table II:** Levels of Anti-pneumococcal IgG to PCV 10 in Different Age Groups

Variables	N	PCV 10 IgG (IU/ml)	Test-statistic	p-value

Age group (months)*			H=5.76	0.22
<12	63	10.90(10.50-11.30)		
12-23	77	10.90(10.60-11.50)		
24-35	40	11.00(10.60-11.00)		
36-47	32	10.90(10.60-11.40)		
48-59	33	11.20(10.70-11.80)		

* Kruskal-Wallis test conducted and results presented in median (IQR)

H: Kruskal-Wallis test statistic

The mean anti-pneumococcal IgG in males was 11.06±1.13IU/ml compared to 10.95±1.35IU/ ml in females, the difference was however not statistically significant (p= 0.48).

## Discussion

The mean anti PCV 10 IgG levels assayed in children who had received three doses of PCV 10, attending the Children Outpatient clinic in Federal Medical Center Owerri, Nigeria was high. This infers that PCV 10 is highly immunogenic and agrees with studies done by Odusanya et al^12^ among Nigerian children and Dicko et al^13^ among Malian children. Similar results were obtained in studies done in Taiwan 16, The Netherlands^17^ and Chile^18^. The antibody response has been good across various geographical regions and there are no studies with contrary findings. The results obtained from this study and similar ones, confirm the immunogenicity of PCV 10, and validate the studies done during the pre-vaccination trials.^5^,^19^

All the participants in the study population formed adequate antibodies to PCV 10. Dicko et al13 in a randomized control trial done in Mali, showed that 97.2% of children formed protective levels of anti-pneumococcal IgG following 3 doses of PCV 10 at 6, 10 and 14 weeks of age. In comparison less than 10% of a control group formed protective antibodies.^13^ Odusanya et al^12^ observed that 98.5 % formed protective levels after PCV 10 immunization. However, the samples were taken after a booster dose.12 Bermal et al^20^, in the Philippines, reported 98.2% after 3 doses of PCV 10. Similar results were reported by Tzou-Yien Lin et al^16^, Lagos et al^18^, and Knuf et al^21^.

In this study, there was a weak positive correlation between the antibody levels following PCV 10 vaccination and the age of the children, as children in the older age group had slightly higher antibody levels. This may be due to the efficacy of the vaccine, in addition to natural immunity developed over time, as nasopharyngeal carriage of pneumococcus is higher in infants and younger children.^22^,^23^ Madhi et al^24^, in a study done in South Africa, determined that after 5.3 years of PCV 9 immunization, some vaccinees had equal or greater portions of the serotype-specific antibodies, compared to the levels assayed in the first year post-vaccination. Other studies have shown that vaccine-elicited pneumococcal antibodies persist for up to 4 years.^25^,^26^ Ekstrom et al^25^, in a Finnish trial, demonstrated that Serum IgG antibody concentrations to vaccine serotypes remained significantly higher in children who had received PCV7 than in control children for 4 years after the fourth PCV7 dose. Wysocki et al^26^ in a study done in 2017 also established that the long-term serotype-specific antibody persistence and robust immunologic memory responses observed suggest induction of long-term protection against pneumococcal disease after PHiD-CV vaccination.26 Similar results were obtained by Prymula et al^27^. On the other hand, Grant et al^28^ observed that PCV serotype-specific IgG concentrations four years following PCV vaccination did not persist above natural levels for most serotypes. The sample size of 32, which was relatively small, compared to others, may have contributed to the divergent result. Further studies are needed to establish the need or otherwise of booster doses of the vaccine.

The anti-pneumococcal IgG concentration for males in this study was slightly higher than that of females, but the difference was not statistically significant. O'Brien et al^29^, demonstrated that sex was not a predictor to antibody response to PCV 10, over the range of their study.^29^ However, Vorsey et al^30^, in a meta-analysis of nine trials among children under three years of age, demonstrated significant differences between girls and boys for diphtheria toxoid, capsular group A, W, Y meningococcal, and pneumococcal vaccines, with immune responses to vaccines consistently higher or equivalent in girls compared with boys. Flanagan et al^31^ observed that females typically develop higher antibody responses and report more adverse effects of vaccination than do males. Flanagan's study was however not specific to the paediatric age group, as the study comprised of children, young adults, and aged individuals.^31^

The conflicting results regarding the relationship between sex and antibody response to PCV 10 may warrant further studies and consideration of biological sex in the planning of clinical trials of vaccines.

The results obtained in this study may be a pointer to the effectiveness of the Nigerian National Programme on Immunization (NPI) at all levels of healthcare. It is recommended that efforts be made to ensure that every child receives three doses of Pneumococcal Conjugate Vaccine 10, at 6, 10, and 14 weeks of age and that the National Programme on Immunization be further strengthened. This should be done by advocacy for more people to enrol, provision of vaccines including vaccine storage facilities, continuous training of health workers and dissemination of information on the effectiveness of the vaccine. A downward trend in the burden of invasive pneumococcal diseases among children in our environment should soon be noticeable.
